# Embryo stage of development is not decisive for reproductive outcomes in frozen-thawed embryo transfer cycles

**DOI:** 10.5935/1518-0557.20170007

**Published:** 2017

**Authors:** Bruno R de Carvalho, Marina W Paes Barbosa, Helena Bonesi, David B Gomes Sobrinho, Íris O Cabral, Antônio C Paes Barbosa, Adelino A Silva, José R Iglesias, Hitomi M Nakagawa

**Affiliations:** 1 GENESIS - Center for Assistance in Human Reproduction, Brasília, DF, Brazil

**Keywords:** Embryo development, reproductive outcomes, frozen-thawed embryo transfer, blastocyst

## Abstract

**Objective:**

To evaluate if the outcomes of IVF/ICSI in frozen-thawed embryo transfer and fresh embryo transfer cycles differ in relation to cleavage and blastocyst stages.

**Methods:**

Retrospective cohort study to compare IVF/ICSI outcomes between fresh embryo transfer and frozen-thawed embryo transfer cycles, according to the stage of embryo development. Analysis was carried out on 443 consecutive embryo transfer cycles performed between January 1st and December 31st, 2014. Women aged up to 38 and submitted to embryo transfer cycles with fresh (n = 309) or frozen-thawed (n = 134) embryos at a private center for assistance in human reproduction were considered for analysis. Results in each group were stratified according to the stage of embryo development: cleavage stage and blastocyst stage. Main outcome measures were implantation rate, clinical pregnancy rate, ongoing pregnancy rate and live birth rate per cycle.

**Results:**

In the fresh embryo transfer group, for cleavage stage versus blastocyst stage, respectively, implantation rates were 22% and 47% (*p* = 0.0005); clinical pregnancy rates were 34% and 64% (*p* = 0.0057); the ongoing pregnancy rates were 30% and 61% (*p* = 0.0046) and live birth rates were 28% and 55% (*p* = 0.0148). There were no significant differences in the rates between cleavage and blastocyst stages in the frozen-thawed group, neither between fresh and frozen-thawed cleavage embryo transfers nor between fresh and frozen-thawed blastocyst transfers.

**Conclusion:**

Our results confirm that blastocyst transfer is better than cleavage stage in fresh embryo transfer cycles. In frozen-thawed cycles, cleavage or blastocyst stages seem to offer similar reproductive outcomes.

## INTRODUCTION

In Assisted Reproductive Techniques (ART), successful implantation depends not only on embryo quality, but also on endometrial receptivity (Evans *et al*., 2014). Traditionally, embryo transfers (ET) were performed on the cleavage stage, because the uterus was supposed to be the best environment for embryo survival (Laverge *et al*., 2001). On the other hand, understanding embryo metabolism and development of sequential culture media enabled embryo transfers on blastocyst stage (Gardner *et al*., 1998 a,b).

Theoretically, blastocysts present a higher implantation potential, since *in vivo* embryos reach the uterus at least on day 4 of fertilization (Gardner *et al*., 1996). There is also evidence that uterine contractility decreases at the time of blastocyst transfer, thus reducing the chance of embryo expulsion (Fanchin *et al*., 2001). Finally, after controlled ovarian stimulation, supraphysiological levels of estrogen may alter endometrial receptivity; blastocyst transfer would prevent premature exposure to an altered uterine environment (Gardner *et al*., 1998a,b; Valbuena *et al*., 2001; Fatemi & Popovic-Todorovic, 2013; Roque *et al*., 2015).

This is also the reason for performing frozen-thawed ET. Since endometrial development is more precisely controlled in those cycles, it would be possible to prevent an embryo-endometrium asynchrony (Roque, 2015). However, clinical pregnancy rates (CPR) and live birth rates (LBR) do not seem to differ between frozen-thawed ET and fresh ET cycles, mainly if blastocysts are transferred.

The aim of this study was to evaluate if the outcomes of IVF/ICSI in frozen-thawed embryo transfer and fresh embryo transfer cycles differ according to the stage of embryo development.

## MATERIALS AND METHODS

This was a retrospective cohort study to compare IVF/ICSI outcomes between fresh ET and frozen-thawed ET, according to the stage of embryo development (cleavage versus blastocyst). The analysis was carried out on 443 consecutive ET cycles performed between January 1st and December 31st, 2014. The main outcomes were implantation rate, clinical pregnancy rate, ongoing pregnancy rate and live birth rate per cycle.

We studied the women aged up to 38 and submitted to ET cycles with fresh (n = 309) or frozen-thawed (n = 134) embryos at a private center for assistance in human reproduction. Results in each group were stratified according to the stage of embryo development: cleavage stage (from day 2 to day 4) and blastocyst stage (days 5 and 6). Table 1 presents patients' characteristics per treatment group. The evaluated outcomes were biochemical pregnancy, clinical pregnancy, ongoing pregnancy and live birth rates.

**Table 1:** Patients' characteristics per treatment group.

Outcomes	Cleavage	Blastocyst	*p* value^a^
*Fresh ET*			
Age, years	33.9 ± 3.25	33.03 ± 2.26	NS
Embryos transferred, n	2.04 ± 0.52	1.94 ± 0.35	NS
*Frozen-thawed ET*			
Age, years	32.9 ± 3.96	31.64 ± 4.02	NS
Embryos transferred, n	2.05 ± 0.49	1.85 ± 0.42	0.0486

The data is expressed as means ± standard deviations

ET = embryo transfer; NS = not significant

a Statistical analysis performed by unpaired t-test or Mann-Whitney test or Fisher's exact test

The Institutional Clinical Committee approved the study. Patients gave written consent for assisted reproduction technology treatment, and an oral consent was obtained for confidential data use for research purposes. A specific written informed consent was not considered necessary for this study, since the data was obtained exclusively from patient files, respecting anonymity.

The statistical analysis was performed using GraphPad Prism software, version 5.00 (GraphPad Software, Inc, 2007). Fisher's exact test was used to compare rates between groups. The level of significance was set at *p* < 0.05 in all analyses.

## RESULTS

In the fresh ET group, for cleavage stage versus blastocyst stage, the implantation rates were 22% and 47% (*p* = 0.0005), respectively; clinical pregnancy rates were 34% and 64% (*p* = 0.0057), ongoing pregnancy rates were 30% and 61% (*p* = 0.0046) and live birth rates were 28% and 55% (*p* = 0.0148). There were no significant differences in rates between cleavage and blastocyst stages in the frozen-thawed ET group (Table 2).

**Table 2:** Comparison of reproductive outcomes from fresh and frozen-thawed embryo transfers, according to embryo development stage.

Outcomes	Cleavage (%)	Blastocyst (%)	*p* value a	OR	95% CI
***Fresh ET***					
Implantation	22	47	0.0005	0.3209	0.1712- 0.6018
Clinical pregnancy	34	64	0.0057	0.2918	0.1231- 0.6918
Ongoing pregnancy	30	61	0.0046	0.2730	0.1150- 0.6482
Live births	28	55	0.0154	0.3268	0.1385- 0.7712
***Frozen-thawed ET***					
Implantation	27	39	NS	0.5691	0.2991- 1.083
Clinical pregnancy	43	53	NS	0.66	0.2855- 1.526
Ongoing pregnancy	36	45	NS	0.6878	0.2928 - 1.616
Live births	36	40	NS	0.8187	0.3467- 1.933

Outcomes presented: Implantation: the ratio of the number of fetal hearts to the number of embryos transferred; Clinical pregnancy: an ultrasound confirmed fetal heart after the 6th gestational week; Ongoing pregnancy: pregnancy continued after the 14th gestational weekET = embryo transfer; NS = not significant; OR = odds ratio; CI = confidence interval

a Statistical analysis performed by unpaired t-test or Mann-Whitney test or Fisher's exact test

## DISCUSSION

Our results confirm that blastocyst transfer is better than cleavage stage ET in fresh cycles. Our findings are in agreement with both systematic reviews assessing this comparison, which favored blastocyst culture for improving live birth rates (Papanikolaou *et al*., 2008; Glujovsky *et al*., 2012). The most likely explanation is that blastocysts may be able to overcome the negative impact by controlled ovarian stimulation on endometrial receptivity (Gardner *et al*., 1998a,b; Valbuena *et al*., 2001). Besides, it seems that there is a natural selection through embryonic development, which does not allow most of the chromosomally abnormal embryos to reach higher developmental stages, and ultimately to implant (Gardner *et al*., 1998 a,b; Papanikolaou *et al*., 2008).

On the other hand, in frozen-thawed ET cycles, cleavage or blastocyst stages seemed to offer similar reproductive outcomes. This was probably because the non-stimulated endometrium (or minimally stimulated) in those cycles may be highly effective in equalizing the results for embryos at any stage of development.

There are several factors that explain why supraphysiological steroid levels impair embryo implantation. First of all, controlled ovarian stimulation (COS) leads to advancement of pinopodes appearance and histological features linked to implantation, resulting in an embryo-endometrium asynchrony (Mirkin *et al*., 2004). There is also a progesterone receptor down-regulation in glandular and stromal cells, leading to a desynchrony, where glandular and stromal maturation do not match the same day (Valbuena *et al*., 2001; Mirkin *et al*., 2004).

Another point is the evidence that COS causes deregulation of more than 200 genes related to implantation, in comparison to natural cycles. The altered gene expression profile leads to an aberrant endometrium, which probably impairs the ideal intrauterine microenvironment for embryo implantation (Horcajadas *et al*., 2005; Labarta *et al*., 2011).

Moreover, elevated estrogen concentration may increase uterine contractions. This effect is more important for cleavage ET, since the contractility decreases progressively, reaching a nearly quiescent status at the time of the blastocyst transfer (Fanchin *et al*., 2001).

Based on the obtained data, we were not able to find significant differences in rates between fresh and frozen-thawed cleavage ET or between fresh and frozen-thawed blastocyst ET. Previous studies have demonstrated similar results for cleavage stage embryos (Bdolah *et al*., 2015), but conflicting results have been published regarding blastocyst transfers (Feng *et al*., 2012; Doherty *et al*., 2014; Özgür *et al*., 2015; Gomaa *et al*., 2016).

The existence of biases related to the retrospective design of our study cannot be excluded and our results must be considered with caution. Statistical analysis demonstrated a small but significant difference in the numbers of embryos transferred between frozen-thawed cleavage and blastocyst transfers; thus, implantation rates may be misleading (Griesinger, 2016). Also, our study did not explore the number or even the existence of top quality embryos at the freezing, thawing and transfer times, which are supposed to be factors that may negatively influence results. There is recent evidence that transferring one top quality embryo may be more important to improve the chance of a live birth than endometrial function in frozen-thawed cycles (Veleva *et al*., 2013), and it may be related to a higher PR (Salumets *et al*., 2006).

Besides, our study design limited exploration of other factors that may influence reproductive outcomes, such as body mass index, etiopathogenic diagnosis of infertility, primary versus and secondary infertility, pregnancy in a previous fresh cycle, type of luteal support, precocious progesterone elevation.

In conclusion, our study suggests that blastocysts are the best option for fresh ET. However, in frozen-thawed ET, no significant differences in reproductive outcomes were found between cleavage and blastocyst stages.
